# Comparison between the effects of one-day treatment regimen with cisplatin on renal function and various biochemical parameters in patients with gastric and lung cancer compared with two-days divided cisplatin treatment regimen

**DOI:** 10.12861/jrip.2015.17

**Published:** 2015-09-01

**Authors:** Ahmad Ahmadzadeh, Heshmatollah Shahbazian, Neda Safapour, Mehri Tulabi, Sepideh Zandifar

**Affiliations:** ^1^Department of Hematology, Ahvaz Jundishapur University of Medical Sciences, Ahvaz, Iran; ^2^Chronic Kidney Disease Research Center, Faculty of Medicine, Ahvaz Jundishapur University of Medical Sciences, Ahvaz, Iran; ^3^Department of Neurology, Ahvaz Jundishapur University of Medical Sciences, Ahvaz, Iran

**Keywords:** Cisplatin, Nephrotoxicity, Kidney

## Abstract

**Introduction:** Cisplatin is an efficient chemotherapeutic drug used for the treatment of different cancers. Clinical trials represents cisplatin-induced nephrotoxicity in a dose dependent manner.

**Objectives:** This study aimed to compare the effects of 1-day treatment regimen with cisplatin on renal function, potassium, calcium, magnesium and uric acid in patients with gastric and lung cancer compared with 2-day divided cisplatin treatment regimen to suggest appropriate management for decrease nephrotoxic effects and >electrolyte abnormalities.

**Patients and Methods:** The study was conducted as a randomized clinical trial. The sample consisted of 60 patients with gastric and lung cancer treated with cisplatin in Ahvaz Shafa hospital. Patients were randomly divided into 2 equal groups. Both groups were treated with cisplatin over a period of 6 to 18 weeks. The first group received 50 mg/m^2^/day of cisplatin during one day and the second group received 25 mg/m^2^/day in 2 days. Electrolytes in each period and renal function at baseline and 6 months after starting treatment was assessed.

**Results:** Difference of mean of renal function and nephrotoxicity incidence in the 2 groups was statistically significant. The odds of hypokalemia in 1-day group was higher than 2-day group (odds ratio [OR] = 6.5), which was statistically significant. However there was no significant relationship between the types of treatment and the risk of hypocalcemia, hypomagnesemia and hyperuricemia. Conclusion: The result of this study showed that, the divided administration of cisplatin reduces the nephrotoxic and hypokalemia effects of this drug, however it had not significant influence on hypomagnesemia, hypocalcaemia, and hyperuricemia.

**Implication for health policy/practice/research/medical education:**

Two of the most serious toxic effects of cisplatin are nephrotoxicity and electrolyte abnormalities. Therefore, assessment of renal function in patients treated with cisplatin in the early stages of treatment is necessary to prevent permanent damage to the kidneys. In a study, we found that, the divided administration of cisplatin reduces the nephrotoxic and hypokalemia effects of this drug, however it had not significant effect on hypocalcaemia, hypomagnesemia and hyperuricemia.

## Introduction

Cisplatin is a strong and effective chemotherapeutic drug used for the treatment of cancers, including sarcoma and certain types of cancer (e.g. small cell lung cancer), lymphoma and germ cell tumors (1). Early clinical trials of cisplatin represent serious toxic effects including gastrointestinal toxicity, neurotoxicity, ototoxicity, and nephrotoxicity (2). The therapeutic effects of cisplatin significantly increases with increasing its dose; however, limits its therapeutic efficacy due to nephrotoxic effects (2,3). Cisplatin-induced nephrotoxicity in humans and animals has been observed in a dose dependent manner (3). Free cisplatin filters within glomeruli (80% of the administered dose is excreted in the urine within 24 hours). Renal blood flow decreases during three hours after cisplatin infusion and decrease in glomerular filtration rate (GFR) subsequently occurs (4). Cisplatin accumulates in the kidneys and its nephrotoxic effect is proportional to the amount of drug accumulation in kidney (5). According to initial reports, for one to 2 weeks after treatment, around 25% of patients who received a single dose of cisplatin had been involved reversible azotemia. Irreversible renal failure (requires dialysis) occurs due to prescribe a higher dose of medication or multiple treatment cycles (2,5,6). The incidence and severity of the renal toxicity increase with frequent use of chemotherapy with cisplatin and consequently causing irreversible damage to the kidneys. Risk factors for the incidence of nephrotoxicity of cisplatin include high levels of plasma free platinum concentration, previously received cisplatin, previous kidney disturbance, the concomitant use of other nephrotoxic substances (1). An analysis of 400 patients by de Jongh et al (6), to determine risk factors for nephrotoxicity, reported that older age, being a smoker, being a woman, hypoalbuminemia, concomitant use of Paclitaxel, radiation to the kidneys and alcohol use (4) are concomitant with the incidence of nephrotoxicity induced by cisplatin.

Risk of nephrotoxicity can be minimized by changing in medication dosing. Unfortunately, most clinical trials have failed to determine reduction dosage for drugs that are excreted by the kidneys in patients with impaired renal function, hence, to prevent renal toxicity, many physicians experimentally reduce the dose (7).

Renal toxicity due to cisplatin induces the pathological changes in urinalysis and serum electrolytes and reduction of renal function. Therefore, assessment of kidney function in patients treated with cisplatin in the early stages of treatment is necessary to prevent permanent damage to the kidneys (8).

One of the most common complications of treatment with cisplatin is the loss of electrolytes and hypomagnesemia. Hypomagnesemia is the most common chronic proximal tubule toxicity and is due to impaired reabsorption of magnesium from the proximal tubule (9). Hyponatremia, hypokalemia and hypocalcemia were observed in some patients. Electrolyte imbalances are common in these patients, but not severe. In the severe cases an electrolyte imbalance can cause ototoxicity and neurotoxicity and the intensification of nephrotoxicity. These conditions can be controlled by supplementation (5).

Cisplatin-induced nephrotoxicity cannot be prevented through traditional methods such as specific process of hydration (1,3), reducing the drug dosage (5,7) and active screening for renal disease. There is not a common recommendation to treat nephrotoxicity (5).

## Objectives

With regard to the nephrotoxic effects of cisplatin, we aimed to compare the effects of 1-day treatment regimen on GFR, potassium, calcium, magnesium and uric acid in the patients with gastric and lung cancer compared with the 2-day divided cisplatin treatment regimen and suggest appropriate management for reduction nephrotoxic effects and electrolyte abnormalities due to cisplatin.

## Patients and Methods

### Patients

This study was conducted as a randomized clinical trial and a blind random sampling (without informing patients of their treatment regimen). The population included all patients with gastric and lung cancer treated with cisplatin in Ahvaz Shafa hospital. The inclusion criteria were age over 18 years, stomach or lung cancer and treatment with cisplatin. Patients receiving other nephrotoxic drugs or with a history of prior chemotherapy, cisplatin dose less than 50 mg/m^2^, dialysis or kidney transplant patients and patients with a GFR less than 70 ml/min were excluded. Patients were divided randomly into 2 groups of 30 patients. Each group received 6 cycles of cisplatin over 18 weeks. In each cycle, the first group regimen was 50 mg/m^2^/day of cisplatin, and in the second group the therapeutic regimen was in the form of administration of 25 mg/m^2^/day of cisplatin in 2 days. According to the usual protocol of chemotherapy, in both groups, patients received hydration with normal saline. Electrolytes in patients at the beginning of each course of chemotherapy and GFR (as a baseline) and 6 months after treatment was evaluated. Data collected included age, sex, history of underlying chronic kidney disease, hypertension, and diabetes, proportion of GFR and measurement of serum levels of creatinine, potassium, calcium, magnesium and uric acid. At the beginning of treatment (as a baseline) and six months after treatment in 2 groups GFR and electrolytes were evaluated. In this study, GFR was measured using modification of diet in renal disease (MDRD) formula.

### Ethical issues

The research followed the tenets of the Declaration of Helsinki. Informed consent was obtained. The research was approved by the Ethical Committee of Ahvaz Jundishapur Medical University.

### Data analysis

The data obtained were encoded and entered the SPSS. The mean ± SD was used to summarize the data and *t* test and logistic regression was used to compare variables in each group. *P* < 0.05 was defined as statistically significant.

## Results

In this study, 60 patients with gastric and lung cancers (33 cases with gastric cancer and 27 cases with lung cancer) were enrolled in 2 groups of 30 patients. During the study, three patients in 2-day group and one patient in 1-day group died and were excluded. The baseline characteristics of both groups are listed in Table 1.

**Table 1:** Basic data in both groups before the study

	**One-day group**	**Two-day group**	***P***
Age (y)	60.10 ± 8.872	61.80 ± 8.438	0.45
Sex (male/female)	53.3%/46.7%	40%/60%	0.3
CKD	13.3%	6.7%	0.67
HTN	16.7%	20%	0.73
DM	13.3%	20%	073
Serum creatinine (mg/dl)	0.69 ± 0.12	0.67 ± 0.12	0.6
GFR (ml/min)	96.6 ± 10.24	98.97 ± 8.49	0.3

Abbreviations: CKD, Chronic kidney disease; HTN, Hypertension; DM, Diabetes mellitus;GFR,Glomerular filtration rate

In *t* test analysis carried out in 1-day and 2-day groups the following results obtained. Nephrotoxicity was seen in 6 patients (22%) in 1-day group and in 2 patients (6%) in 2-day group (*P* = 0.025).

In 1-day group the mean GFR at the end of treatment was 78.17±16.584 and in 2-day group was 88.87 ± 12.921 min/ml (*P* = 0.007).

In 1-day group the mean ± SD serum creatinine was 0.89 ± 0.23 mg/dl and in 2-day group it was 0.79 ± 0.15 mg/dl. Therefore, according to the *P* = 0.07 there was no statistical significant difference between 2 groups (Table 2).

**Table 2:** GFR and Cr in the 2 groups by *t* test at the end of treatment

	**One-day group**	**Two-day group**	***P***
GFR (ml/min)	78.17±16.584	88.87±12.921	0.007
Cr (mg/dl)	0.89±0.23	0.79±0.15	0.07

Abbreviations: GFR,Glomerular filtration rate; Cr, creatinine.

Then multivariate analysis was performed using logistic regression model and the following results were obtained. Chance of hypokalemia in 1-day group was 6.5 times the 2-day group (CI: 1.820-23.213; *P* = 0.004).

The odds of hypocalcaemia in 1-day group was 7.3 times of the 2-day group (CI: 0.815-64.457); *P* = 0.076), which there was not statistically significant difference, however it was marginal.

The odds of hypomagnesemia in 1-day group was 4.5 times of the 2-day group (CI: 1.094-18.503; *P* = 0.37). The odds of hyperuricemia in 1-day group was 3.2 times of the 2-day group (CI: 0.316-32.889; *P* = 0. 32) (Table 3).

**Table 3:** Odds ratio and CI in the 1-day group versus 2-days group about electrolyte disorders

**Electrolyte disorder**	**Odds ratio**	**95% CI**	***P*** ** value**
Hypokalemia	6.5	1.820-23.213	0.004
Hypocalcemia	7.3	0.815-64.475	0.076
Hypomagnesemia	4.5	1.094-18.503	0.37
Hyperuricemia	3.2	0.316-32.889	032

## Discussion

The first mechanism in creating nephrotoxicity by cisplatin is cell toxicity. Inside the cells in an environment containing low chloride, parts of cisplatin chloride are replaced by water molecules. It was found that the products resulting from the hydrolysis reaction show a reaction with cytoplasm glutathione and DNA (10). The molecular events lead to stop dividing of malignant cells. By this mechanism the nephropathy is created by cisplatin, which is a common complication. Second mechanism is renal vasoconstriction that causes reduced renal blood flow where the event occurs immediately after the injection of cisplatin. Third mechanism is pro-inflammatory effects of this drug. Cisplatin increases the secretion of inflammatory cytokines such as TNFα, Interferon gamma (IFNγ), and caspases that these stimulate differentiation, maturity, and activation of neutrophils and T cells and other components of the inflammatory response (11,12). The potential importance of these mediators during acute renal failure with less intense was seen in the kidney tissue of mice that were exposed to cisplatin (13,14). The fourth mechanism is an effect on proximal tubules. Cisplatin causes selectively necrosis and death of proximal tubule cells, although the cells that are not dividing are generally less sensitive to the toxicity of DNA-damaging agents (15).

Few studies have described the pathology associated with cisplatin nephrotoxicity in humans. The site of injury is distal tubules and collecting ducts and proximal tubule. The difference in the effect may be due to differences in the duration and dose of medication. About 3-60 days after drug administration, biopsies collected represent a segmental degeneration, necrosis and destruction of the proximal tubule and distal tubule and collecting ducts, respectively (16).

The more important presentation of cisplatin nephrotoxicity has impaired renal function, which can be progressive. Other protests that have been described are hypomagnesemia, loss of minerals, Fanconi-like syndrome and microangiopathic anemia. According initial reports, one to 2 weeks after treatment approximately 25% of patients who receive a single dose of cisplatin has been restored reversible azotemia.

Irreversible renal failure (in need of dialysis) occurs with multiple or high administration of the medication dosage or treatment cycle (2,5,6). The incidence and severity of the nephrotoxicity increase with the frequent use of chemotherapy with cisplatin and consequently causing irreversible damage to the kidneys. Nephrotoxicity occurs commonly with bolus injection of cisplatin that it can be split up over several days to reduce the effects, although enough studies have not been carried out in this area. In a study conducted on mice, in single-dose regimen, in the third day, blood urea nitrogen (BUN) and Cr increased while the divided regimen had not effect on the biochemistry. In the this research the extensive histological degenerative and regenerative epithelial changes were observed following the administration of cisplatin in 1-day while by giving the divided regimen and single dose had similar behavior on kidney but for divided regimen the changes on the kidney tissue was very lower.

In the present study to evaluate the nephrotoxic effects of divided 2-day treatment with cisplatin, 60 patients with gastric cancer and lung cancer in 2 groups, with a mean age of 60.10 ± 8.872 years and 61.80 ± 80438 years were enrolled in 1-day and in 2-day groups, respectively.

In the 1-day group nephrotoxicity was seen in six patients (22%) and in the 2-day group in 2 patients (6%) was seen, therefore, according to the *P* = 0.025, there was statistically significant difference. There was not a significant difference of GFR at baseline between groups. However, at the end of the treatment, a significant difference between a decline in GFR in 2 groups was detected (*P* = 0.007). Additionally, about the effects of cisplatin on serum electrolytes, the following results were obtained: the risk of hypokalemia in one-day was 6.5 times the 2-day group (CI: 1.820-23.213), hence, there was a statistically significant difference.

Chance of hypocalcaemia in 1-day group was 7.3 times the 2-day group (CI: 0.815-64.457), while, the difference was not significant, however, it was marginal (*P* = 0.076). Risk of hypomagnesemia in 1-day group was 4.5 times the 2-day group (CI: 1.094-18.503), which was not statistically significant.

Hyperuricemia chance in 1-day group was 3.2 times the 2-day group, which was not statistically significant.

A study was conducted by the English et al (17), in England in 1999 to assess the renal function changes, time of the changes, and to identify risk factors for nephrotoxicity in children treated with carboplatin. The glomeruli and proximal tubule function was assessed in 23 patients receiving carboplatin before treatment and after 2 years of treatment. Average decline in GFR was 22 ml/min/1.73 m^2^ (*P* = 0.012) and serum magnesium concentration was 0.17 mmol/l (*P* = 0.0077), which was statistically significant, however, GFR and serum magnesium levels did not change after treatment. It seems the results of the current study are consistent with results of the study that had been done by English et al in the short-time but long-time follow-up of patients is necessary (17). Likewise, in a study by Erdlenbruch et al (18), in 2001 to evaluate the relationship between the pharmacokinetics of cisplatin and nephrotoxicity on 12 children showed that long-term infusion of cisplatin has a less nephrotoxicity effect than other methods of intermittent bolus administration of cisplatin in patients. In this study, the relationship between the maximum concentration of cisplatin in plasma and urine were associated with the lowest point of GFR (*P* = 0.05). The results of this study are consistent with the present study.

A prospective longitudinal study at a center in New Castle by Skinner et al (9), was conducted to assess the long-term nephrotoxicity of cisplatin and carboplatin in 63 children and adolescents treated with platinum compounds (cisplatin 27, 24 carboplatin and 12 both drugs) from 1981 to 1996. The evaluation was done at the end of one year and 10 years later. No significant change was observed in any of the treatment groups (treated with cisplatin, carboplatin or both) during 10 years in renal function. The lack of consistency with the results of the current study appears to be due to the short duration of our study.

A descriptive study by Fatima et al (8) in 2004-2005 on renal function of 36 patients with different types of cancer that received CDDP (cis-diamminedichloroplatinum), GFR was estimated before and after administration of six cycles of CDDP (the higher dose of 250 mg/m^2^). The mean reduction in GFR at baseline and after 6 cycles was 43.86 ml/min/1.73m^2^ (*P *< 0.001), which was statistically significant and consistent with the results of the current study.

In order to evaluate the effect of cisplatin, etoposide and bleomycin on long-term kidney function a retrospective study was done by Suer et al (19), from 1995 to 2013 of 157 patients with germ cell testicular tumors that 113 patients were receiving chemotherapy. During the follow-up visit, significant differences were observed in serum creatinine and estimated GFR in patients between groups with chemotherapy and no chemotherapy.

Significant decrease in GFR (*P* = 0.001) and a significant increase in new onset of chronic kidney disease stage 3 were seen in the group receiving chemotherapy with cisplatin as compared with patients without chemotherapy. This study is consistent with our results.

In a study conducted by Yoshida et al (20), in 2014 about the prophylactic effect of magnesium on cisplatin nephrotoxicity, 496 patients with thoracic malignancies were treated with 260 mg/m^2^ cisplatin during January 2009 to December 2011 and according to the results of the study nephrotoxicity was 19.1% in the first cycle and 39.7% at the end cycle. The results of this study also indicated a high probability of occurrence of cisplatin nephrotoxicity in daily regimen, which is consistent with our results.

In clinical trials conducted by Anvari et al (21), in 2010, entitled “the assessment of magnesium supplementation in the prevention of hypomagnesaemia caused by cisplatin,” 59 patients treated with cisplatin in oncology center hospital, Mashhad, were evaluated. This study showed that the incidence of hypomagnesaemia in patients receiving cisplatin as a single dose is higher than divided drug recipients (4.71 vs. 9.42% of the *P* = 0.056) which is consistent with our study.

## Conclusion

The results of this study showed that the divided administration of cisplatin reduces the nephrotoxic and hypokalemia effects due to this drug, however, it had not significant effect upon hypocalcemia, hypomagnesemia and hyperuricemia. Next question is whether the division of cisplatin into 2 days affects the quality of the treatment or not. Further studies are needed to answer this question. This study was intended merely for treatment of renal complications.

## Limitation of the study

Our study had some limitations as follow:

1. Low number of samples
2. Sample reduction from the study due to the put-off or death
3. Short duration of study.

## Authors’ contribution

NS and SZ prepared the primary draft. HS and AA revised the manuscript. MT further edited the paper. All authors read and signed the paper.

## Conflicts of interest

The authors declared no competing interests.

## Ethical considerations

Ethical issues (including plagiarism, misconduct, data fabrication, falsification, double publication or submission, redundancy) have been completely observed by the authors.

## Funding/Support

This article was extracted from thesis by Neda Safapour for internal medicine residential thesis. This study was supported by a grant from Ahvaz Jundishapur Medical University (grant No. UN1229).
